# Heat treatment and its effect on machining induced surface roughness of cast and additive manufactured AlSi10Mg

**DOI:** 10.1038/s41598-025-10732-5

**Published:** 2025-07-21

**Authors:** Amith K. V., Satish Shenoy Baloor, Ashwin Polishetty, Gururaj Bolar, Arun N. Govindhan

**Affiliations:** 1 Department of Mechanical and Industrial Engineering, Manipal Institute of Technology, Manipal Academy of Higher Education, Manipal, Karnataka 576104 India; 2 Department of Aeronautical and Automobile Engineering, Manipal Institute of Technology, Manipal Academy of Higher Education, Manipal, Karnataka 576104 India; 3 School of Engineering, Computer and Mathematical Science, Auckland University of Technology, Auckland, 1010 New Zealand; 4 Oerlikon Balzers Coating India Private Limited, Tumakuru, Karnataka 572128 India

**Keywords:** AlSi10Mg, Metal additive manufacturing, DMLS, Post-processing, Surface roughness, Cleaner production, Heat treatment, Engineering, Materials science

## Abstract

The study investigates the post-printing machinability of AlSi10Mg aluminum alloy, with a primary focus on evaluating T6 heat treatment influence on the cutting force and surface quality of additive manufactured material while comparing the outcomes with the cast alloy. Milling experiments were performed on untreated and heat-treated specimens using a range of cutting speeds and feed rates. Results revealed that the surface roughness of as-cast AlSi10Mg alloy was 50.5–532.3% higher than that of the as-printed material. This was attributed to the coarser microstructure and lower microhardness (73.04 HV) of the as-cast alloy, which led to increased plastic deformation during machining, leading to increased surface roughness, especially at lower feed rates. In contrast, the fine cellular microstructure of the as-printed alloy enhanced the microhardness (140.3 HV) and deformation resistance, producing better surface quality. T6 heat treatment significantly affected the surface roughness of the cast and AM aluminum alloy. While heat treatment enhanced the surface finish of cast alloy, it reduced the surface roughness in the printed alloy. T6 heat treatment refined the microstructure of cast and printed alloys, increasing microhardness (126.1 HV) and reducing plastic deformation in cast alloy while reducing the microhardness (78.7 HV) and increasing the ductility of the printed alloy, potentially increasing the surface roughness. Despite the treatment, surface roughness of heat-treated cast alloy remained 19.3–38.2% higher than that of the heat-treated printed material. Cutting force analysis showed that additively manufactured (AM) specimens experienced a 49.5–178.1% increase in cutting force compared to as-cast specimens. However, when heat-treated, the AM specimens exhibited a 16.9–67.1% reduction in cutting force relative to untreated AM parts. In contrast, heat-treated as-cast specimens showed a moderate increase in cutting force, ranging from 17.1 to 56.3%, compared to their untreated counterparts. The findings emphasize the significant influence of material manufacturing route and heat treatment on the machining outcomes. The results show that the section on milling process variables depends on the material fabrication route and heat treatment applied during the post-processing stage.

## Introduction

Metal Additive Manufacturing (AM) is an advanced production method that builds metallic components layer by layer. This technology has gained significant attention due to its efficiency, competitiveness, and ability to produce intricate, high-quality, near-net shape products^1^. Over the past decade, AM has experienced rapid growth and commercial success across various industries, including automotive, aerospace, marine, and biomedical^2,3^. The process supports a spectrum of materials, including steels, titanium, aluminum, nickel, and cobalt alloys. Among these, aluminum alloys rank as the second most widely used after steels, owing to their lightweight nature, high specific strength, excellent corrosion resistance, and superior thermal and electrical conductivity^4,5^. Notably, AlSi10Mg, a silicon-rich near-eutectic (hypoeutectic) aluminum alloy, stands out in AM applications due to its exceptional flowability and resistance to cracking during solidification^6,7^. This makes it a preferred choice for architectural design and tooling, where its short solidification range, excellent printability, and cost-effectiveness facilitate rapid prototyping and efficient manufacturing^7^.

Despite the advantages of metal AM technology in terms of flexibility and efficiency, the AM parts may not be ready for immediate service due to poor dimensional accuracy and surface quality. Furthermore, AM parts may exhibit suboptimal mechanical properties in their as-built state due to anisotropic behavior, residual stresses, and microstructural defects^8,9^. Post-processing techniques are employed to refine microstructure, enhance mechanical properties, and improve surface finish and dimensional accuracy^10^. Heat treatment plays a vital role as a post-processing method, helping optimize the microstructure and enhance the mechanical performance of metal parts for practical applications. Heat treatment facilitates phase transformations, grain refinement, and stress relief by precisely controlling heating and cooling parameters, enhancing mechanical performance.

Several studies have explored the microstructure and post-printing heat treatment of AlSi10Mg to improve its mechanical properties. During AM, AlSi10Mg is subjected to rapid heating, cooling, and solidification cycles, resulting in a fine cellular α-Al phase surrounded by fibrous Si network^11,12^. This ultrafine microstructure contributes to increased hardness and strength. However, when subjected to T6 heat treatment, the microstructure coarsens, reducing the grain boundary strengthening effect^12,13^. As a result, grain boundary density decreases, facilitating dislocation motion, reducing hardness^12,13^, and improving ductility^14,15^. However, this increase in ductility comes at the cost of reduced ultimate tensile strength^11,15^. Additionally, T6 heat treatment reduces material anisotropy and alleviates residual stresses^15^. Aging AlSi10Mg without solution heat treatment has been shown to enhance strength by forming stable nanosized Si precipitates^16^. Furthermore, heat treatment effectively lowers residual stresses without significantly altering the fine cellular structure of SLM AlSi10Mg^11,17^.

The cooling method used during heat treatment also influences mechanical properties. A comparative study of water quenching, air cooling, and furnace cooling revealed that rapid cooling promotes a finer microstructure, increasing hardness, whereas slow furnace cooling leads to Si coarsening and spheroidization, thereby reducing the hardness^12^. Similarly, fracture behavior studies of AlSi10Mg subjected to T5, standard T6, and rapid T6 heat treatments found that early material failure was due to voids and fragmentation of the silicon network. T6-treated specimens exhibited enhanced ductility as a result of globular morphology of Si particles, which reduced the stress concentration. Rapid T6 treatment yielded finer, more homogenous Si particles, further enhancing ductility over standard T6 treatment^18^. However, solution heat treatment has also been shown to enlarge gas pores, reducing material density and negatively impacting mechanical properties^19^.

Post-processing operations, such as machining, are essential for improving the surface finish dimensional accuracy of AM parts. The machinability of AM AlSi10Mg differs from that of cast AlSi10Mg due to its refined microstructure. Studies have shown that AM AlSi10Mg generates higher cutting force and lower surface finish compared to cast AlSi10Mg under similar cutting conditions, primarily due to its fine cellular dendritic microstructure^20^. However, this fine microstructure also increases material hardness, reducing milling-induced burrs in AM specimens^20^. Material properties also influence chip formation. AM AlSi10Mg produces shorter chips than its cast counterpart, as its higher porosity and brittleness lead to increased microcrack formation and enhanced chip breakability^21^. Machinability comparisons between cast and AM A205 aluminum have shown that AM components exhibit higher Specific Cutting Energy (SCE) and vibrations, but their surface roughness remains largely unaffected. Due to their smaller grain size and higher microhardness, AM components often achieve a better surface finish than their cast counterparts^22^. Heat treatment further influences machinability; as-built and solution-treated AM aluminum alloys, with their higher ductility, exhibit poorer surface finish, increased vibrations, and higher SCE^23^.

Researchers have explored several strategies to enhance the machinability of AM AlSi10Mg. Down-milling has been identified as a preferred machining approach, improving surface finish compared to conventional milling methods^21^. The choice of cooling techniques greatly affects the surface finish, tool life, and cutting temperatures. Among dry cutting, flood cooling, and Minimum Quantity Lubrication (MQL), MQL-assisted machining significantly improves surface quality, reducing surface roughness by 45–63% compared to dry cutting and by 23–43% compared to flood cooling. Additionally, MQL minimizes tool wear, reducing flank wear by 29–45%, and lowers the cutting temperatures when compared to dry and flood-assisted machining^24^. The use of vegetable oil in MQL further reduces machining temperature by 24–39% and tool wear by 18–31%, offering a superior surface finish over flood cooling^25^. Cryogenic cooling has also emerged as an effective alternative, outperforming dry, flood, and MQL-assisted machining by significantly reducing tool wear^26^.

The reviewed literature clearly indicates that post-print machining is essential for enhancing the surface quality of AM parts. Additionally, AM alloys possess unique microstructural and mechanical properties compared to conventionally manufactured materials, which can significantly influence their machinability. However, existing research lacks a comprehensive comparison of how process variables affect the surface quality of cast and AM AlSi10Mg. Using machining parameters optimized for conventional materials on AM components may lead to inferior surface integrity and excessive tool wear. Additionally, previous studies have not provided a direct comparison of the machinability between heat-treated cast and AM AlSi10Mg. To address this gap, the current research investigates the machinability of AlSi10Mg alloy by evaluating surface roughness and cutting force. The study further evaluates the impact of T6 heat treatment on cutting force and surface quality of AM AlSi10Mg and compares its performance with the cast AlSi10Mg alloy in its untreated and heat-treated states.

## Materials and methods

### Fabrication of AlSi10Mg parts

**Fig. 1:**
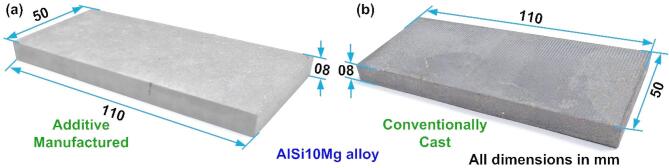
(**a**) AM AlSi10Mg specimen, (**b**) Cast and shaped AlSi10Mg specimen.

The AM AlSi10Mg alloy specimens measuring 110 mm × 50 mm × 8 mm, were fabricated on an EOS M280 Direct Metal Laser Sintering (DMLS) system in an Argon atmosphere. Figure 1a shows the printed AM specimen. The material was developed using gas-atomized powders whose average size ranged between 15 and 50 μm. The AM AlSi10Mg specimens were built with a 370 W laser, 1300 mm/s scan speed, 0.19 mm hatch distance, and 30 μm layer thickness. A stripe scan strategy with a 67° inter-layer rotation^27^ and a stripe width of 7 mm was employed during printing. The cast specimens were manufactured by melting the aluminum alloy in a steel crucible at 740 °C for four hours. Molten metal was cast in a preheated steel mold at a melt temperature of 710 °C. The casting was extracted upon achieving the requisite cooling, and the ingots were subsequently sectioned utilizing Wire Electrical Discharge Machining (WEDM) to attain the precise dimensions necessary for the investigation, as seen in Fig. 1b. The average density of the DMLS AlSi10Mg was 2.68 g/cm³, compared to 2.77 g/cm³ noted for the as-cast AlSi10Mg. Table 1 provides the chemical composition of the alloy.

**Table 1:** Chemical composition of AlSi10Mg alloy.

Material	Elements	Si	Mg	Mn	Cu	Fe	Ni	Zn	Sn	Ti	Pb	Al
AM	Wt%	10.0	0.4	0.4	0.05	0.5	0.05	0.1	0.05	0.15	0.05	Bal
Cast	Wt%	10.7	0.45	0.3	0.03	0.3	-	-	-	0.18	-	Bal

### Heat treatment

The cast and AM specimens underwent T6 heat treatment in three stages. Initially, specimens underwent solution heat treatment at 540 °C for two hours in a muffle furnace. Following the solution treatment, specimens were water-quenched for 3–4 min to prevent any premature precipitation during cooling. After quenching, the specimens underwent artificial aging at 180 °C for six hours in the same furnace. Finally, the specimens were allowed to cool under ambient air conditions.

### Machining experiments

**Fig. 2:**
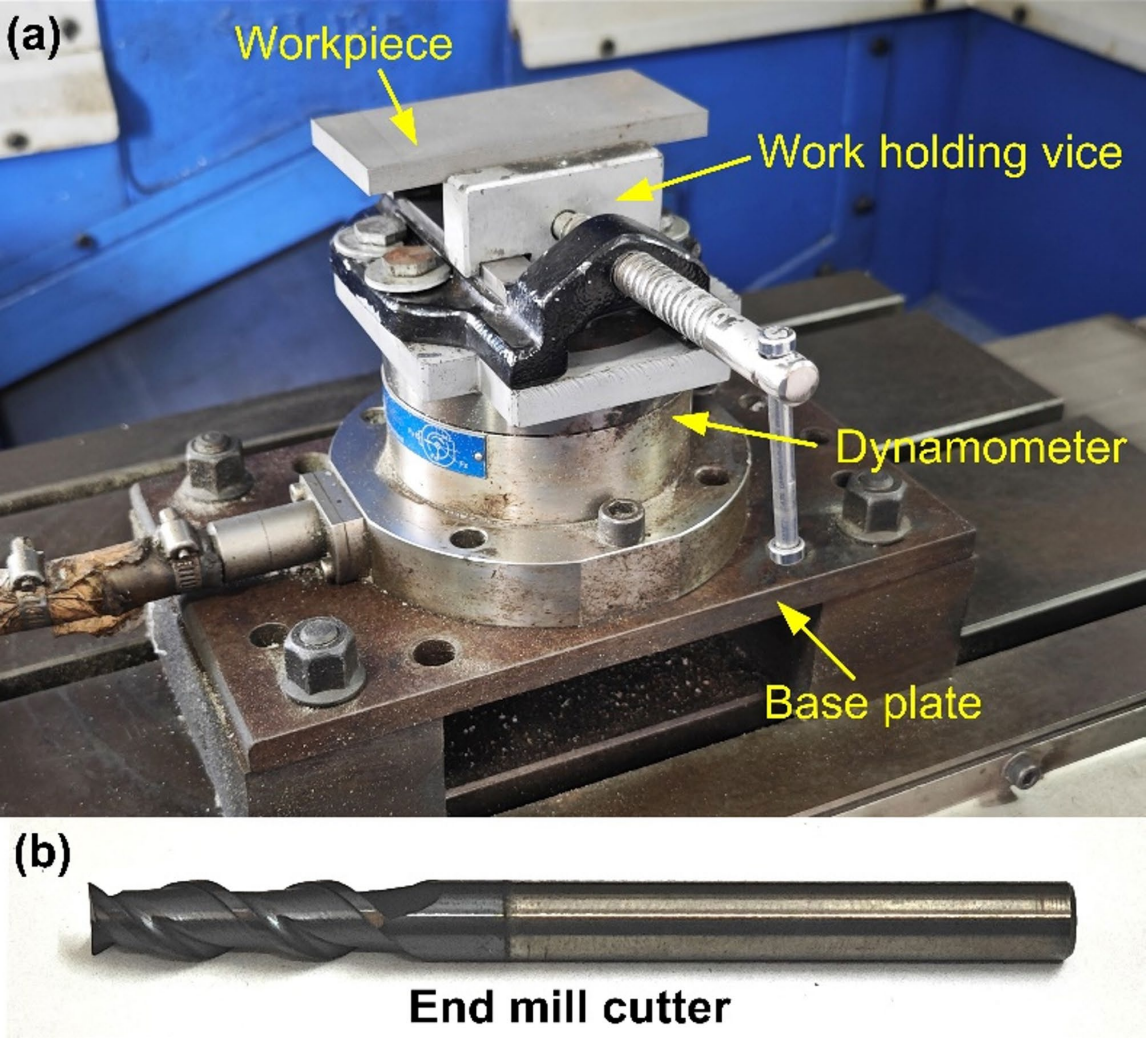
(**a**) Experimental setup for slot milling, (**b**) Coated end milling cutter used during machining experiments.

The machining studies were performed on four types of samples: as-cast, as-printed (AM), T6 heat-treated cast alloy, and T6 heat-treated AM alloy. The milling trials were conducted using a three-axis vertical machining center (VMC) (*AMS Spark*). The machining experiments utilized the fixture shown in Fig. 2a, while Fig. 2b displays one of the coated end mills used during the machining experiments. The end mills were coated with amorphous tetrahedral carbon (ta-C) using PVD-arc technology (*Oerlikon Balzers*). This coating exhibits low friction coefficient (0.1–0.2) and high hardness (50–60 GPa), ensuring excellent tribological performance and enhanced wear resistance. The machining and cutting tool parameters are detailed in Table 2. Each combination of experimental conditions was tested three times to ensure repeatability and consistency of results.

**Table 2:** Slot milling conditions and cutting tool parameters.

Parameter	Machining conditions
Cutting speed	25, 50, 75 m/min
Feed	0.06, 0.12, 0.18 mm/rev
Axial depth of cut	1 mm
Radial depth of cut	5 mm
Milling tool diameter	5 mm
Flutes	2
Helix angle	35°
Flute length	18 mm
Lubrication method	Dry cutting

### Characterization methods

Microstructural characterization of the cast and AM specimens was performed using an optical microscope (*Olympus BX53M*) and scanning electron microscope (SEM) (*Zeiss EVO 18*) equipped with energy dispersive spectroscopy (EDS) probe (*Oxford*) for elemental analysis. Prior to microstructural observation, the specimens were etched in Keller’s reagent, following the ASTM E407 standard. The microhardness of the as-cast and AM AlSi10Mg specimens was evaluated using a Vickers microhardness tester (*Matsuzawa MMT-X*) equipped with a diamond-shaped indenter. A 200 g load was applied for 15 s during each indentation, following the ASTM E92 standard. The milled surface roughness was analyzed using a non-contact surface roughness tester (*Contour-Bruker*). Surface roughness was quantified using the arithmetic mean height (*Sa*) as the measurement unit. Cutting force was measured using a three-component dynamometer (*Kistler 9272*), connected to a charge amplifier (*Kistler 5070 A*) and a data acquisition system. The sampling frequency was maintained at 4000 Hz. Acquired force signatures were processed using *Dynoware* software. The cutting force from the three milling force components *F*_*x*_, *F*_*y*_ and *F*_*z*_ was calculated using Eq. 1^28^.

1 $$F=\sqrt {F_{x}^{2}+F_{y}^{2}+F_{z}^{2}}$$

## Results and discussion

The post-machining surface quality of a workpiece is critical, as it directly influences wear resistance, fatigue strength, and corrosion resistance, which are essential factors for the performance and longevity of any component. Therefore, this study systematically analyzed the milled surface quality of cast and AM AlSi10Mg in both untreated and heat-treated conditions.

### Influence of machining parameters on surface roughness

**Fig. 3:**
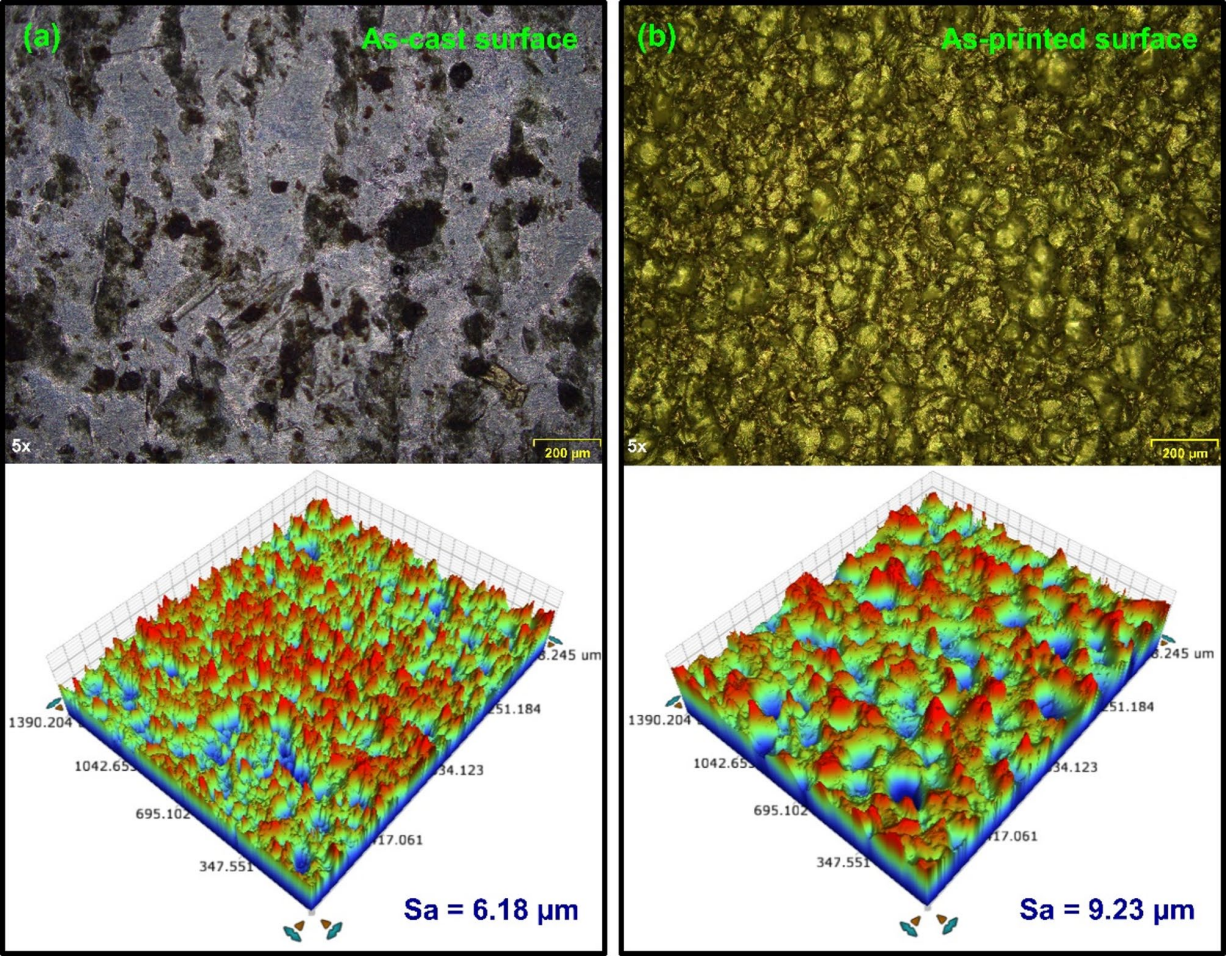
Surface morphology and 3D topography of (**a**) As-cast material, (**b**) As-printed material.

**Fig. 4:**
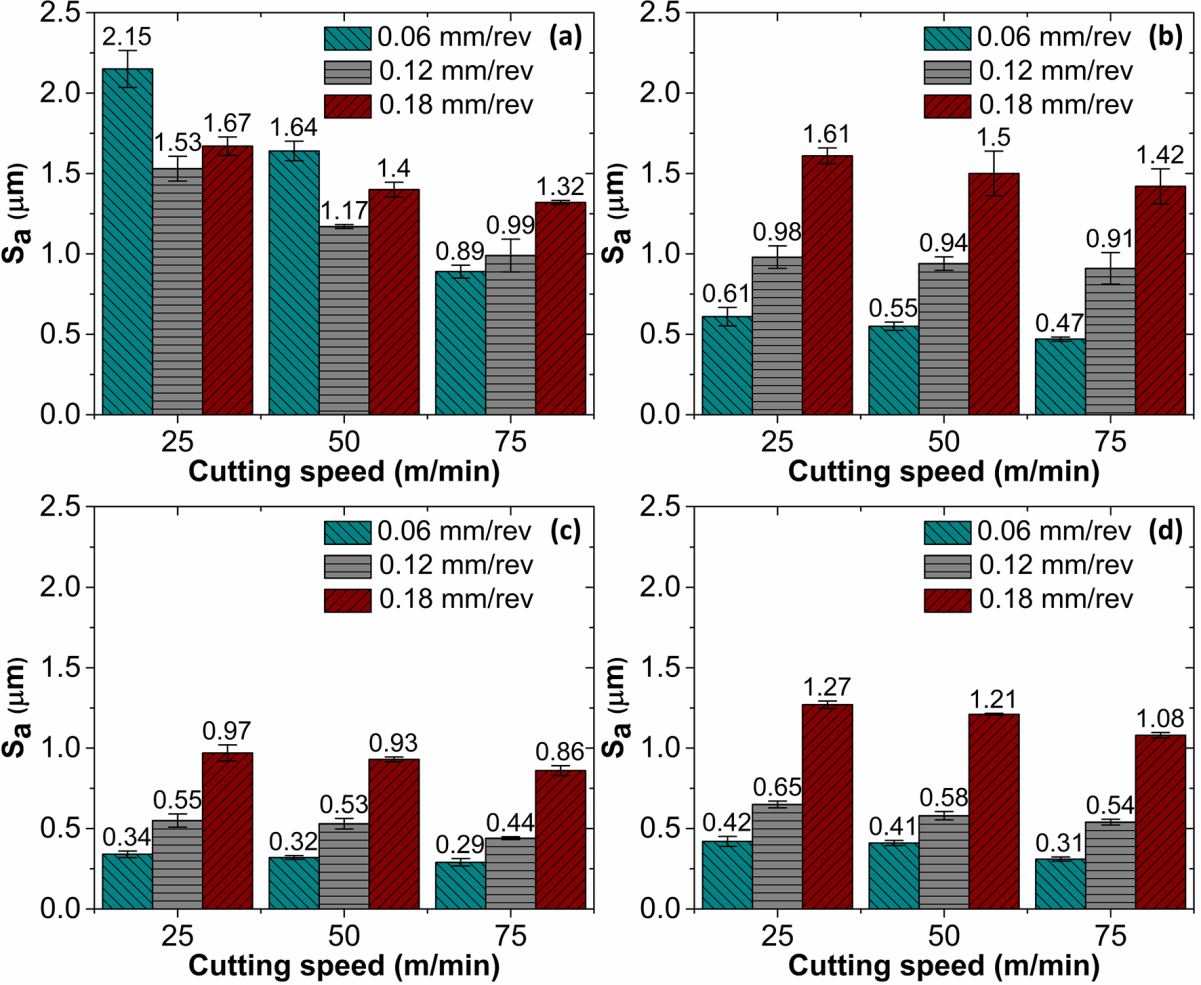
*Sa* variation with process variables for (**a**) As-cast alloy, (**b**) Cast alloy with heat treatment, (**c**) As-printed alloy, (**d**) Printed alloy with heat treatment.

The 3D surface topographies of the as-cast and AM AlSi10Mg surfaces, revealing a significantly high *Sa* is shown in Fig. 3. The average *Sa* obtained while milling cast and AM AlSi10Mg in untreated and heat-treated state with various feed and cutting speed combinations is presented in Fig. 4. With the exception of the untreated as-cast specimen, the *Sa* value increased as the milling feed rate increased. The rise in the *Sa* with the rise in the feed is attributed to chip formation mechanics^29^. During machining, tool cutting edge creates a series of patterns on the workpiece surface, with the spacing between the patterns determined by the selected feed per tooth. As a result, the topography presents itself as a replica of the tool’s leading-edge profile, making surface roughness a function of feed rate. Therefore, at higher feed rates, the tool covers a greater distance per revolution, leaving more widely spaced feed marks on the machined surface, thereby increasing *Sa*. As the feed increases, the initially blunt peaks observed at lower feed rates undergo a transition, forming more pronounced patterns with sharper peaks. This effect increases the height and width of the surface peaks^30^.

Furthermore, a greater volume of material is removed within the same time interval, requiring higher forces to deform the material and exceed its yield strength. Consequently, the cutting force increases as the feed rate rises. This can result in tool deflection and vibration, thus deteriorating the surface finish.

Figure 4 also reveals the impact of cutting speed on *Sa* values of the cast and AM AlSi10Mg under both untreated and heat-treated conditions. A clear trend is revealed where increasing speed reduces the *Sa* values. The improvement in surface finish with higher speed selection can be attributed to several interrelated factors. Higher speeds intensify tool–workpiece friction, producing more heat. However, despite the increased temperatures, higher speeds facilitate smoother material flow and improve chip formation, reducing plastic deformation during chip removal. Excessive heat buildup is avoided by effective heat dissipation at higher speeds, which could otherwise degrade surface quality. Additionally, higher cutting speeds shorten the contact time between the tool and workpiece, enhancing chip evacuation and resulting in a better surface finish. Furthermore, when speed increases, cutting forces decrease, minimizing vibrations and deformation, which contributes to an improved surface texture.

### Influence of material production route on surface roughness

**Fig. 5:**
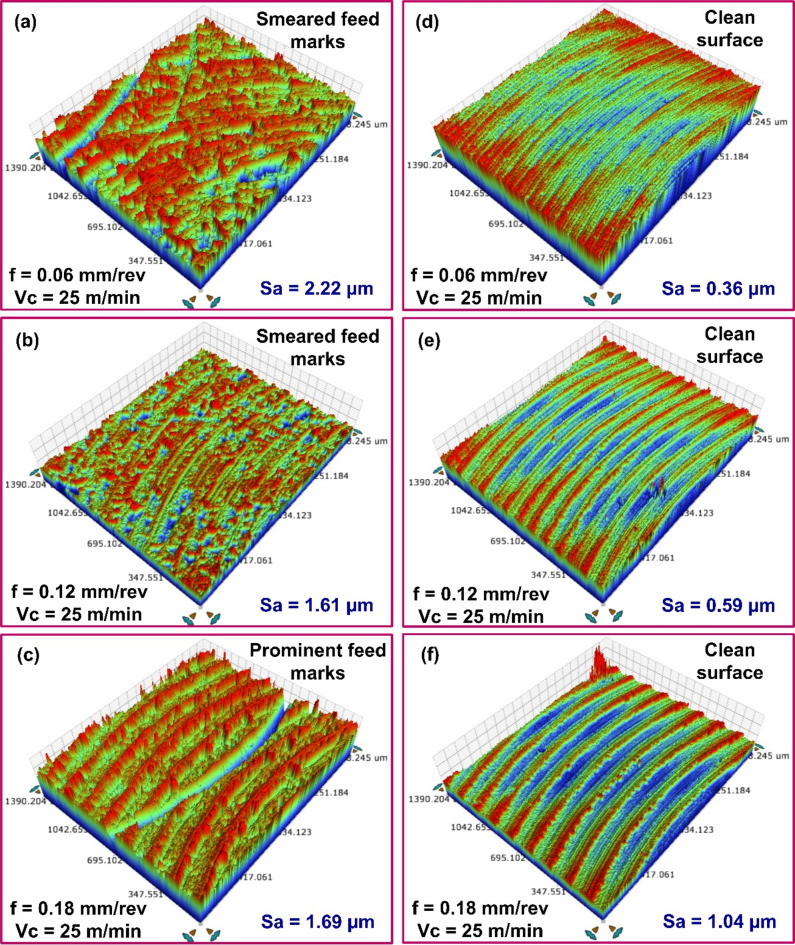
3D topography of milled surfaces with different feed values for (**a**-**c**) As-cast AlSi10Mg, (**d**-**f**) As-printed AlSi10Mg.

Surface roughness varied significantly depending on the material choice. The processing route (Casting and Additive Manufacturing) employed to fabricate the material influenced the microstructure, thereby affecting the surface finish. Notably, higher *Sa* values were observed when milling as-cast specimen at lower feeds. For instance, as seen in Fig. 5(a–c), in as-cast specimens a *Sa* of 2.22 μm was recorded at 25 m/min cutting speed and 0.06 mm/rev feed. Increasing the feed to 0.12 mm/rev reduced *Sa* to 1.61 μm, while further increasing the feed to 0.18 mm/rev led to a slight increase in *Sa* to 1.69 μm. In contrast, the as-printed alloy exhibited significantly lower *Sa* values under similar conditions (see Fig. 5(d–f)). *Sa* of 0.36 μm was recorded with 25 m/min speed and 0.06 mm/rev feed. A higher feed of 0.12 mm/rev resulted in a *Sa* of 0.59 μm, and at 0.18 mm/rev, it further rose to 1.04 μm. Within the given range of process variables, the machining-induced *Sa* for the as-cast AlSi10Mg was 50.5–532.3% higher than as-printed material. These results indicate that the as-cast alloy exhibits poorer machinability compared to the as-printed alloy, as evidenced by its higher surface roughness at lower feeds and cutting speed.

**Fig. 6:**
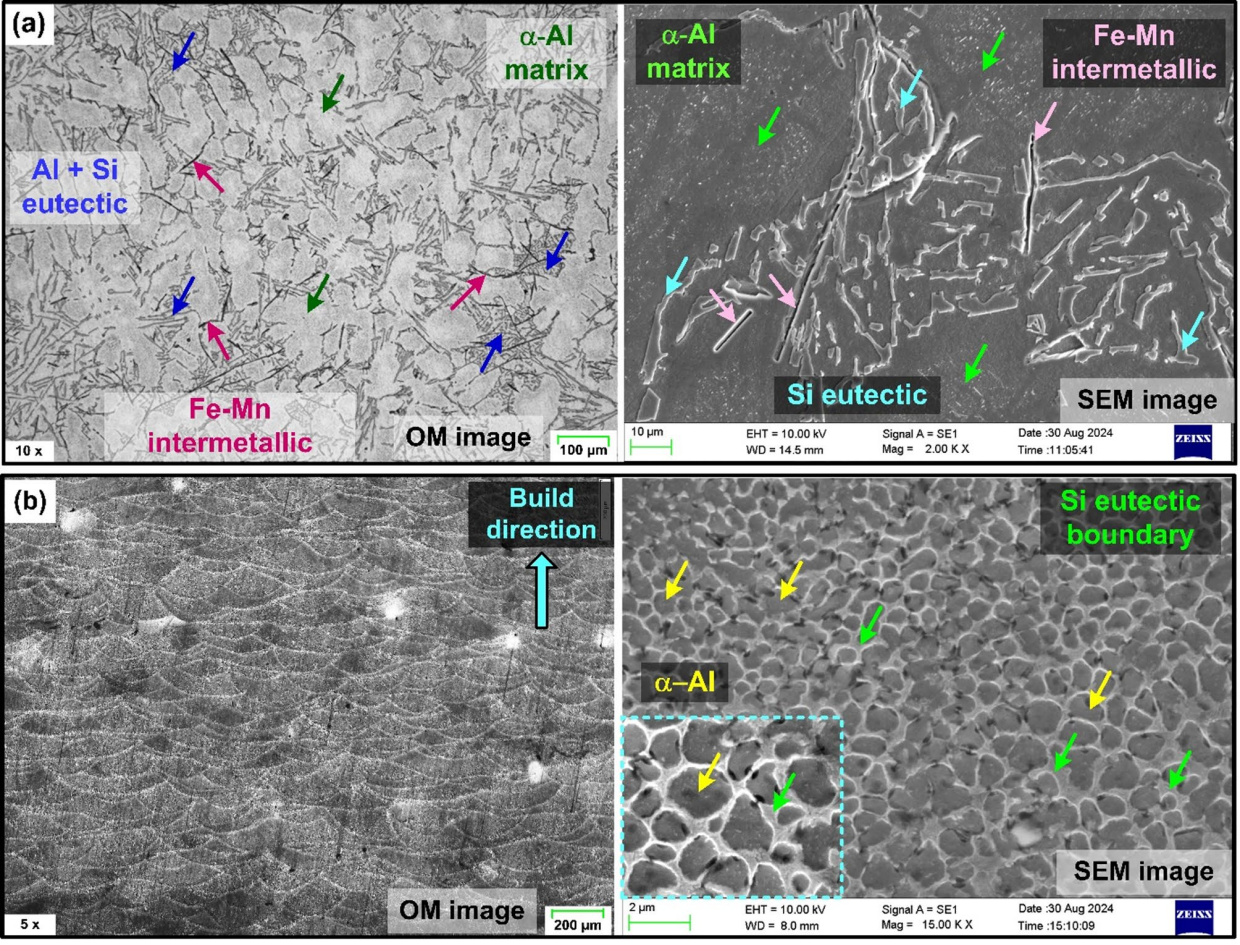
Optical and SEM images showing microstructure of (**a**) As-cast AlSi10Mg, (**b**) As-printed AlSi10Mg.

The poor surface finish of the as-cast alloy, especially at lower feed rates is primarily attributed to material smearing (see Fig. 5a). Microstructure appear to have significantly contributed to the deterioration of surface finish in the as-cast alloy at the lower cutting feed. Slow cooling rate associated with the AlSi10Mg casting processes leads to the disintegration of the silicon (Si) solid solution in aluminum (Al), forming coarser Si precipitates^11,12^. The aluminum matrix, consisting of scattered primary α-Al, is surrounded by a continuous eutectic Al-Si structure as shown in Fig. 6a. Furthermore, the microstructure revealed the presence of dispersed intermetallic like Mg₂Si and needle-like Fe-rich particles. The as-cast AlSi10Mg, whose microstructure is predominantly composed of the α-Al rich matrix exhibits lower microhardness (73.04 HV) and lower yield strength^15^, as compared to the printed material. Therefore, at lower feed rates and cutting speeds, the material undergoes greater plastic deformation, leading to smearing and a poorer surface finish.

Moreover, Warsi et al.^31^ identified specific regions known as ‘avoidance zones’ in metal cutting process. It is a zone where variations in feed and speed can alter the cutting mechanism from shearing to ploughing when machining soft, ductile materials. Within this zone, plastic deformation causes material to drag along the surface, increasing material displacement and side flow, ultimately deteriorating the surface finish. The obliterated feed marks (see Fig. 5a, b) on the machined surface further supports this observation. A similar trend was ascertained when varying feed values at 50 m/min cutting speed. However, at 75 m/min cutting speed, *Sa* followed the typical increasing trend, with increasing with feed rate.

For the given combinations of milling process parameters, the as-printed AlSi10Mg specimens exhibited lower surface roughness than their as-cast counterparts, as illustrated in Fig. 5d–f. Once again, microstructure strongly influenced the roughness of the AM material. As-printed AlSi10Mg specimen displayed a fine cellular dendritic structure formation with fibrous Si particles and cellular α-Al phase surrounding the Al-matrix (see Fig. 6b). A cellular dendritic structure typically forms in metals that solidify at very high cooling rates and low solute concentrations^11,12^. In Al-Si alloys, higher cooling rates increased the solubility of Si in Al. Therefore, DMLS process, known for its very high cooling rates (10³ to 10^8^ K/s), promotes increased silicon solubility in Al-matrix^11,12^. The rapid solidification lowers the Si concentration in the liquid phase, thereby promoting cellular dendritic structure. This fine dispersion of fibrous Si networks in the Al matrix positively influences the microhardness and yield strength of the as-built specimens. According to Li et al.^14^, the ultrafine microstructure with a high density of grain boundaries offers resistance to dislocation motion, leading to dislocation pile-up at grain boundaries. This contributes to increased yield strength and microhardness (140.3 HV) in the AM specimens due to Hall-Petch effect. However, this strength enhancement is achieved by compromising on the material ductility. The reduced ductility had a noticeable effect on machinability. The AM specimens exhibited a more pronounced hardening effect while machining with the milled surfaces of AM parts exhibiting well-defined and consistent feed marks, indicating a superior surface finish compared to the as-cast specimens. Furthermore, as noted by Warsi et al.^31^, AM specimens exited the avoidance zone at lower feed and speeds than their cast counterpart. Accordingly, even at the lowest feed (0.06 mm/rev) and speed (25 m/min) utilized in the presented work, the material removal occurred through shearing mechanism, unlike the ploughing behavior observed in the as-cast specimens. As a result, the AM specimens achieved better surface finish even at lower cutting parameters compared to their as-cast counterparts.

**Fig. 7:**
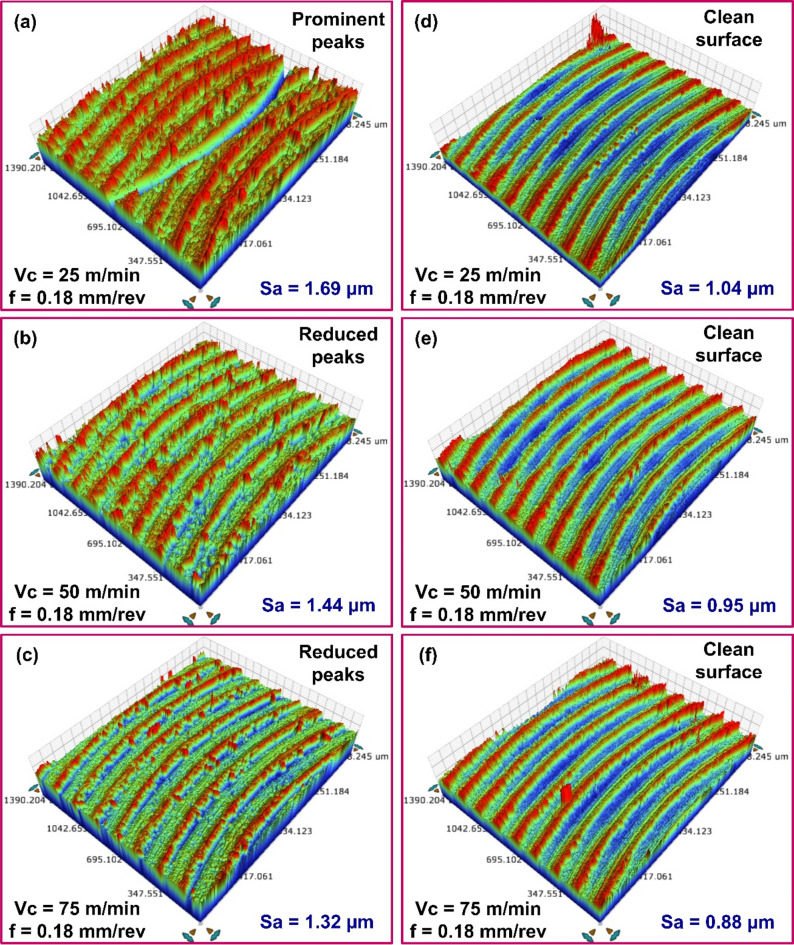
3D topography of milled surfaces with different speed values for (**a**-**c**) As-cast AlSi10Mg, (**d**-**f**) As-printed AlSi10Mg.

The influence of cutting speed on *Sa* during machining processes shows a clear trend. As cutting speed increases, a notable reduction in *Sa* was observed. Impact of cutting speed on *Sa* of the as-cast and as-printed materials is illustrated in Fig. 7a–f. For instance, in as-cast specimens, a *Sa* of 1.69 μm was observed at 0.18 mm/rev feed and 25 m/min cutting speed. Increasing the speed to 50 m/min decreased the *Sa* to 1.44 μm, and a further increase to 75 m/min led to a lower *Sa* of 1.32 μm.

In comparison, the as-printed alloy showed slightly lower *Sa* values under similar cutting conditions. At 25 m/min and 0.18 mm/rev feed, the *Sa* was recorded at 1.04 μm. This value decreased to 0.95 μm at 50 m/min and it dropped to 0.88 μm at 75 m/min. The enhanced surface finish at elevated cutting speeds can be attributed to several interconnected factors. At elevated speeds, excessive plastic deformation generates more heat. Despite the rise in temperature, higher speeds facilitate efficient heat dissipation, minimizing excessive heat accumulation that could degrade surface quality. Additionally, increased cutting speed shortens the work–tool interaction time, enhancing chip evacuation and contributing to a smoother finish. Higher speeds also reduce cutting forces, which in turn lowers vibration and deformation, further improving surface quality. A similar trend was ascertained when varying speed values at feeds of 0.06 mm/rev and 0.12 mm/rev. The results indicate that feed rate had had a greater influence on surface roughness than cutting speed. Using lower feed rates in combination with low cutting speeds during machining of the as-cast material proved detrimental, leading to poorer surface quality. In contrast, combining lower feed rates with higher cutting speeds proved beneficial for both as-cast and printed AlSi10Mg, resulting in cleaner cuts and reduced surface roughness.

### Influence of heat treatment on surface roughness

**Fig. 8:**
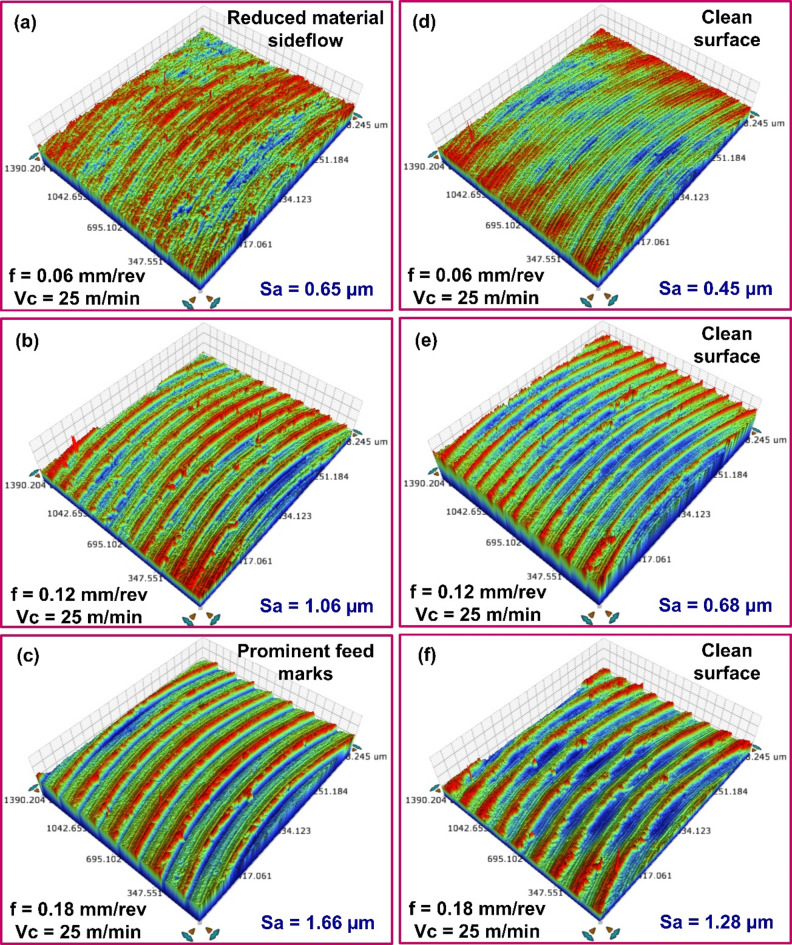
3D topography of surface milled with different feed values for heat-treated (**a**-**c**) Cast AlSi10Mg, (**d**-**f**) Printed material AlSi10Mg.

The heat treatment significantly influences the surface roughness of the cast and AM material. For example, after milling, the heat-treated cast alloy displayed a *Sa* of 0.65 μm with a 25 m/min cutting speed and a 0.06 mm/rev feed. A higher *Sa* of 1.06 μm was measured in specimens milled with 25 m/min cutting speed and 0.12 mm/rev feed. With a further increase in feed to 0.18 mm/rev, the *Sa* rose to 1.66 μm. (see Fig. 8a–c). Following heat treatment, the machining-induced *Sa* of the cast AlSi10Mg was 19.3–38.2% higher than that of the as-printed material. These findings suggest that while heat treatment improved the machinability of the cast alloy, its surface remained rougher than the heat-treated printed counterpart.

**Fig. 9:**
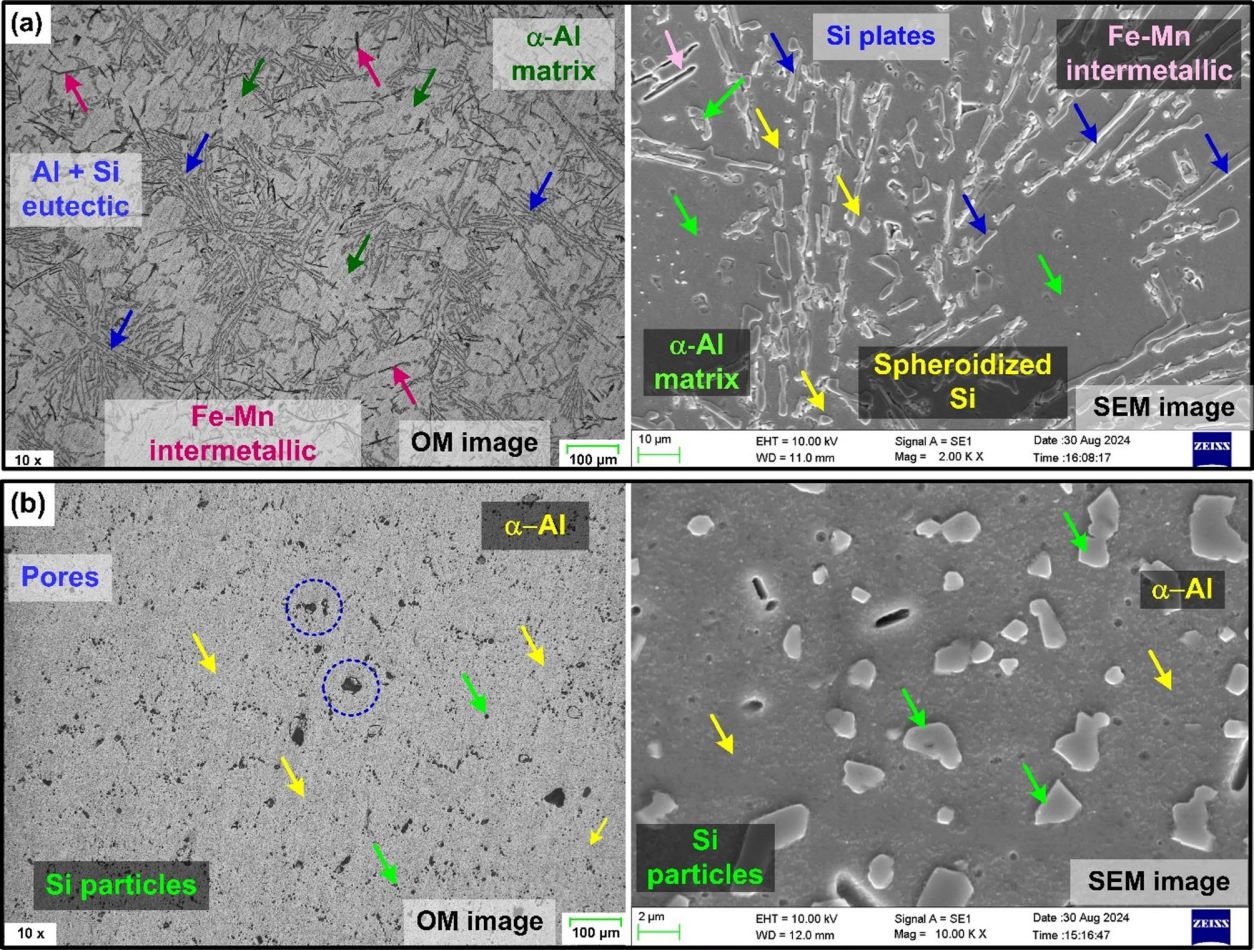
Optical and SEM images showing microstructure of heat-treated (**a**) Cast AlSi10Mg, (**b**) Printed AlSi10Mg.

The enhancement in surface finish of the cast alloy post heat treatment is attributed to several reasons. The as-cast material exhibits a coarser microstructure, which consists of dendritic primary α-Al grains and lamellar eutectic (α-Al + Si) structure, exhibiting lower microhardness and increased plastic deformation tendency, leading to poor surface finish. However, the sharp, acicular Si lamellae with irregular faceted morphology noted in the as-cast condition were transformed into more rounded and disconnected particles following T6 heat treatment (see Fig. 9a). The spheroidization, driven by Ostwald ripening during solution treatment, homogenized the microstructure and reduced the presence of sharp Si particles that could contribute to high adhesion and plastic flow during machining. Moreover, after T6 heat treatment formation of Mg_2_Si precipitates are observed, which in tandem with the intermetallic phases act as a barrier to plastic deformation, increasing the microhardness and yield strength of the cast material^15,32^.

Similar observation was by made by Silva et al.^33^, where the heat treatment of cast AlS10Mg alloy increased the secondary dendrite arm spacing and Si particle size, significantly enhancing the microhardness of the heat-treated specimens. The increase in microhardness (126.1 HV) enhances resistance to plastic deformation and reduces material smearing. As a result, the material removal mechanism shifts from ploughing (for as-cast material) to shearing, due to improved microhardness and deformation resistance after heat treatment. This is evident from the fact that even at lower speed and feed, heat-treated aluminum alloy produced surfaces with clear feed marks, free from material smearing. Moreover, the presence of hard phases reduced material ductility, promoting chip embrittlement and breakability. This, in turn, minimized chip adhesion and build-up edge formation, resulting in an improved surface finish in T6 heat-treated AlSi10Mg compared to the as-cast material under similar cutting conditions.

Heat treatment also influenced the post-machining surface quality of the AM material. The heat-treated AM specimens exhibited higher *Sa* values than their as-printed counterparts (see Fig. 8d–f). A *Sa* of 0.447 μm was recorded in experiments conducted at 25 m/min cutting speed and 0.06 mm/rev feed. At a feed of 0.12 mm/rev, *Sa* of 0.680 μm was observed. At a highest set feed of 0.18 mm/rev, *Sa* of 1.284 μm was noted. Under similar processing conditions, the *Sa* values of heat-treated AM specimens were consistently higher than those of the as-printed material.

Heat treatment causes microstructure coarsening in AM material. As illustrated in Fig. 9b, Si particles in T6-treated specimens become larger and more uniformly distributed due to coalescence and Ostwald ripening. During solution heat treatment, Si particles, initially part of a eutectic structure within the Al-matrix, migrate out due to the supersaturation of Al with Si. As a result, Si particles accumulate along grain boundaries, forming coral-like fibrous structures. These structures then fragment and gradually transform into more rounded shapes through spheroidization^15,34^. Furthermore, the aging process allows the fragmented Si particles from the solution treatment to continue spheroidizing, with enhanced atomic diffusion at elevated temperatures, producing more uniformly distributed, rounded Si particles. Si precipitation from the eutectic network diminishes solid solution strengthening. Eventually, the increased spacing between Si particles reduces the elastic modulus and yield strength of the material^14^. Also, grain boundary reduction due to microstructure coarsening decreases the microhardness (78.7 HV), while enhancing the ductility of AlSi10Mg, post heat-treatment. Consequently, greater plastic deformation occurs during machining, leading to material side flow and increasing the surface roughness of heat-treated AM material.

**Fig. 10:**
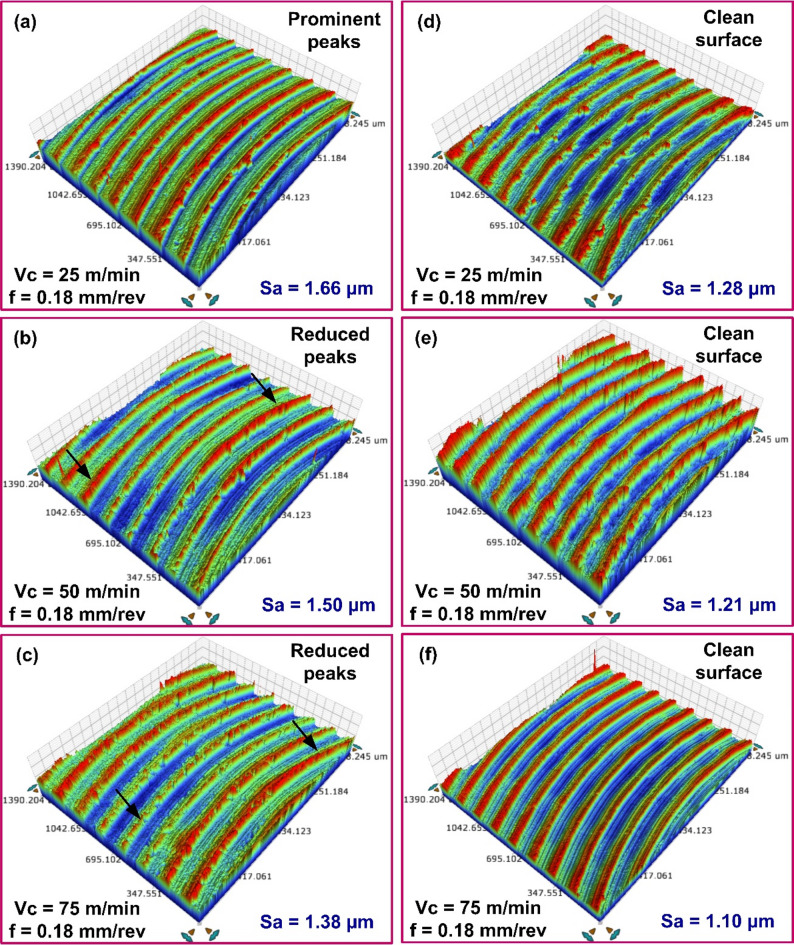
3D topography of surfaces milled with different speed values for heat-treated (**a**-**c**) Cast AlSi10Mg, (**d**-**f**) Printed AlSi10Mg.

Figure 10a–c illustrates the influence of cutting speed on *Sa* of cast specimens subjected to heat treatment. For instance, an average *Sa* of 1.66 μm was recorded at 25 m/min speed and 0.18 mm/rev feed. Increasing the cutting speed to 50 m/min reduced *Sa* to 1.50 μm, and a further increase to 75 m/min lowered it to 1.38 μm. At lower cutting speeds, noticeable adhesion occurred during initial tool engagement, contributing to a rougher surface finish. Increased cutting speeds led to lower cutting forces, primarily due to diminished friction at the tool–chip interface. This can lower the tool deflection and vibration, thus contributing to the improved surface finish. The decreasing trend in *Sa* was also observed across other feed conditions. A similar pattern was noted during the milling of heat-treated printed material. As shown in Fig. 10d–f, at 0.06 mm/rev feed, increasing the cutting speed led to a progressive reduction in *Sa* values. *Sa* of 1.28 μm at 25 m/min, 1.21 μm at 50 m/min, and 1.10 μm at 75 m/min were recorded, indicating improved surface finish with higher speeds. Notably, *Sa* values recorded while milling as-cast specimens were generally higher than those observed for the heat-treated cast alloy. As discussed earlier, this difference is primarily attributed to variations in microstructure and material microhardness. Additionally, post-machining analysis of the heat-treated AlSi10Mg surfaces showed no signs of material adhesion, and surface quality consistently improved with increased cutting speed.

Furthermore, *Sa* values of heat-treated cast and printed specimens exhibit an interesting trend. While they are generally comparable, the heat-treated cast specimens show slightly higher *Sa* values. The reduction in ductility of cast alloy after heat-treatment helped control the plastic deformation and material side flow, thus improving the surface finish. In contrast, for the heat-treated printed alloy, microstructure coarsening during solution treatment reduced grain boundaries, leading to decreased microhardness and increased ductility. Specifically, the coarsening and spheroidization of Si particles, along with reduced mirohardness, contributed to greater plastic deformation during machining. This increased plasticity resulted in material side flow, thus increasing the *Sa* values. Moreover, microstructure of heat-treated AM AlSi10Mg revealed the presence of pores. In additive manufacturing, pores typically form during the material building process due to factors such as melt splashing, hydrogen absorption, or Marangoni flow^35^. This can result in gas entrapment within the material during solidification. During subsequent heat-treatment, the internal gas pressure increases, causing the surrounding material to deform, resulting in the enlargement of existing pores, as illustrated in Fig. 9b. The presence of these enlarged pores negatively impacts the milling process and increase the surface roughness of AM AlSi10Mg post heat treatment.

### Cutting force analysis

**Fig. 11:**
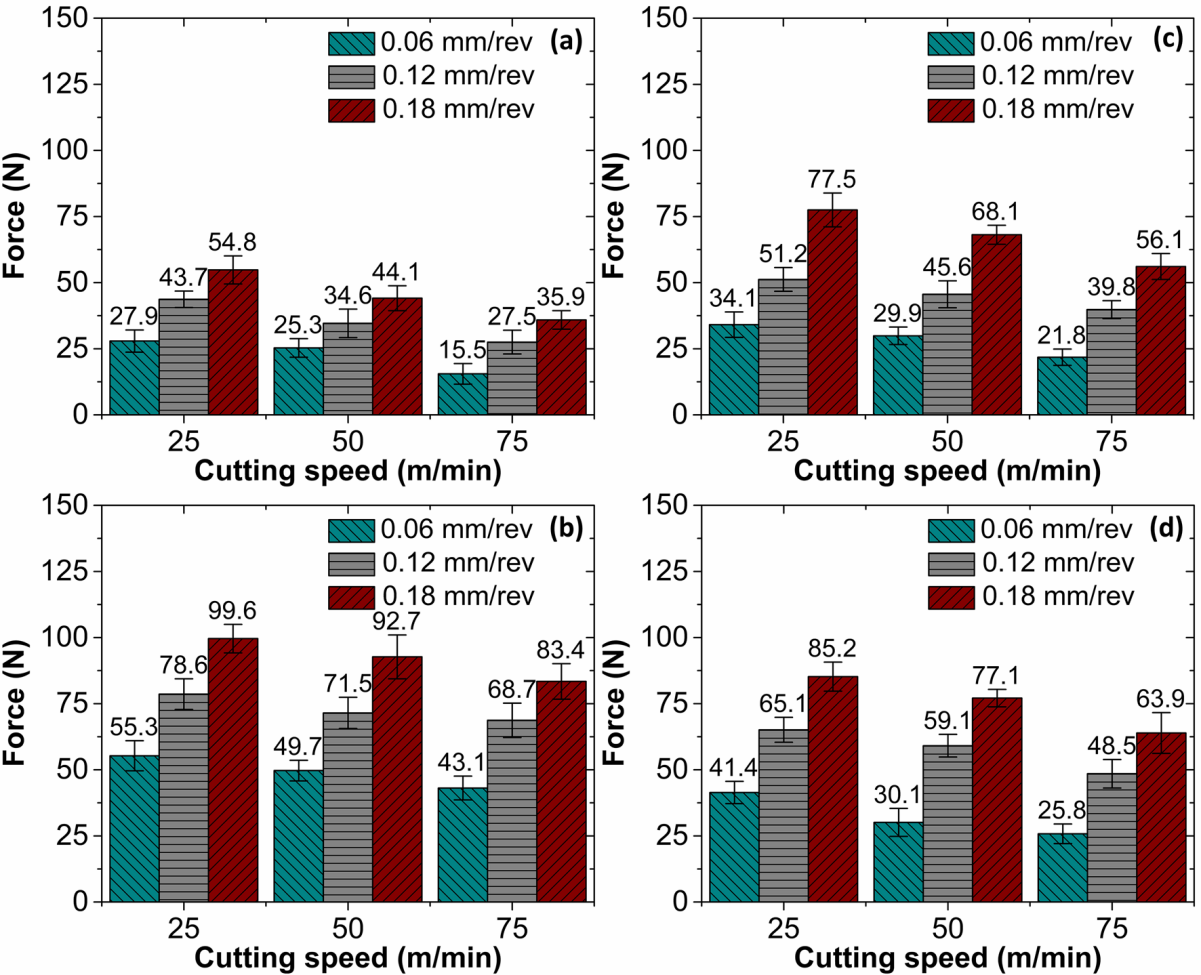
Cutting force variation with process variables for (**a**) As-cast alloy, (**b**) As-printed alloy, (**c**) Cast alloy with heat treatment, (**d**) Printed alloy with heat treatment.

Cutting force is a crucial parameter in the machining process which can help in understanding and optimizing the machining process. Therefore, in the present study the influence of material fabrication route and heat treatment on the cutting force was analyzed. Figure 11 presents the cutting forces measured during milling of cast and AM AlSi10Mg specimens, both in the as-fabricated and heat-treated conditions. Across all four material conditions, cutting force magnitude consistently decreased with increasing cutting speed. This trend is attributed to the increased material deformation rate at higher speeds, which causes the cutting process to become adiabatic. Under adiabatic conditions, limited heat dissipation leads to increased temperatures in the cutting zone. The rise in temperature induces thermal softening of the work material, reducing its resistance to cutting and thereby lowering the cutting forces^36^. Additionally, Fig. 11 shows that cutting forces increase with feed rate in all material conditions. With cutting speed and depth of cut held constant, increasing the feed rate results in a thicker undeformed chip. This increases the volume of material being sheared at the cutting edge, requiring more energy to plastically deform the material and, consequently, generating higher cutting forces^20^.

Additionally, cutting force measured while milling the as-printed AlSi10Mg was higher than the force developed while milling as-cast specimens. For instance, at a cutting speed of 25 m/min and a feed rate of 0.06 mm/rev, the cutting forces measured were 27.9 N and 55.3 N for the as-cast and as-printed specimens, respectively, reflecting a 98.2% increase in the AM condition. Similar trends were observed across other speed and feed combinations. The elevated cutting forces in the AM specimens can be attributed to their fine, anisotropic cellular-dendritic microstructure. Due to rapid solidification during additive manufacturing, the cooling rate is significantly higher than in casting, leading to increased silicon solubility in the aluminum matrix and the formation of a cellular-dendritic microstructure. This refined structure results in increased microhardness (140.3 HV) due to the Hall-Petch effect, where grain boundaries impede dislocation motion^14^. During milling, the ultrafine microstructure resists plastic deformation, resulting in dislocation pile-up and localized strengthening, thereby requiring greater energy and force to machine the material. In contrast, the lower cutting forces observed in the as-cast AlSi10Mg specimens are a consequence of their coarser microstructure and reduced hardness. The slower cooling rate in casting allows for the breakdown of Si solid solution, leading to the formation of coarser Si precipitates^11,12^. The matrix consists mainly of ductile α-Al and a eutectic Al-Si structure, offering less resistance to plastic deformation. This facilitates easier material removal, reducing the required cutting force.

Heat treatment had a notable impact on cutting force behavior. For the cast material, cutting forces increased after heat treatment. At a cutting speed of 25 m/min and feed of 0.06 mm/rev, a 22.2% rise in cutting force was recorded post-treatment. Conversely, heat treatment led to a reduction in cutting force for AM specimens, with a 33.6% decrease as compared to the as-printed specimen. This divergence in behavior is linked to the microstructural changes induced by T6 heat treatment. In cast AlSi10Mg, the treatment promotes spheroidization of acicular Si particles during the aging phase. The rounded morphology of Si particles reduces stress concentrations and enhances hardness. Additionally, small amounts of Fe and Mn, associated with intermetallic particles, remain within the heat-treated alloy, further contributing to the material’s hardness by providing additional barriers to plastic deformation^32^. As a result, the effort needed to machine the material increased, thus increasing the cutting force. However, in the case of AM AlSi10Mg, heat treatment results in microstructural coarsening. The coral-like fibrous Si structures fragment and gradually spheroidize, leading to more uniformly distributed, rounded particles. Furthermore, the fragmented Si particles from the solution treatment continued to spheroidize during the aging process, producing more uniformly distributed, rounded Si particles. Si precipitation from the eutectic network diminishes the solid solution strengthening, thereby increasing the ductility of the material after heat treatment^15^. As a result, the softened microstructure offers less resistance during machining, lowering the cutting force required.

## Conclusions

The cast and AM AlSi10Mg machinability was assessed by examining the effects of different speed-feed combinations on surface roughness and cutting force. Additionally, the effect of heat treatment on cutting force and surface finish was examined. The key conclusions are as follows:

- Microstructure significantly influences the surface quality of machined components. The as-cast alloy exhibited a coarser microstructure with lower microhardness (73.04 HV), resulting in more pronounced plastic deformation during machining and consequently, higher surface roughness. In contrast, the fine cellular microstructure of the as-printed alloy improved microhardness (140.3 HV) and resistance to deformation, leading to a superior surface finish.
- The as-cast alloy displayed greater surface degradation at lower feed rates and cutting speeds. Tool marks and material smearing were more prominent in as-cast material, further compromising the surface quality. Under identical machining conditions, the surface roughness of as-cast AlSi10Mg was found to be 50.5–532.3% higher than the as-printed AlSi10Mg.
- T6 heat treatment altered the microstructure and microhardness of both cast and printed AlSi10Mg. In the cast alloy, it refined the microstructure and increased the microhardness (126.1 HV), thereby reducing plastic deformation. Conversely, in the printed alloy, it lowered the microhardness (78.7 HV) and enhanced the ductility, which contributed to increased surface roughness during machining.
- Post-heat treatment, the surface roughness of machined cast AlSi10Mg was 19.3–38.2% higher than the printed counterpart. Heat treatment improved the surface finish of the cast alloy, while printed alloy exhibited a decline in surface finish compared to the pristine material.
- Cutting force magnitude when milling printed AlSi10Mg was 49.5–178.1% higher the force recorded while milling as-cast material. The higher cutting force is attributed to the ultrafine microstructure and high density of grain boundaries density observed in the printed material. Heat treatment had a notable impact on cutting force. A notable increase in cutting force was observed when milling the heat-treated cast material, while a declining trend was observed in the case of printed AlSi10Mg post heat-treatment. The relative changes in the cutting forces are attributed to the heat-treatment induced microstructural refinement in the cast and printed AlSi10Mg.

Overall, machinability differed significantly between cast and additively manufactured AlSi10Mg. As-printed alloy consistently demonstrated the best surface finish among all tested conditions. This study highlights that the cutting parameters tailored for machining materials fabricated using a casting process cannot be directly applied to the machine the AM material. However, the current work can be extended to explore the influence of heat treatment on other machinability evaluation parameters such as specific energy consumption, chip morphology and cutting temperature in order have a comprehensive understanding of the machinability of AlSi10Mg. Furthermore, the study can further be expanded to assess the influence of heat treatment and material production routes on the machinability of other AM materials.

## Data Availability

“Data is provided within the manuscript or supplementary information files”.
